# Antirheumatic drug leflunomide attenuates atherosclerosis by regulating lipid metabolism and endothelial dysfunction via DHODH/AMPK signaling pathway

**DOI:** 10.7150/ijbs.93465

**Published:** 2024-07-02

**Authors:** Xinhai Jiang, Weizhi Wang, Lijuan Lei, Tingting Feng, Yang Hu, Peng Liu, Yining Li, Ren Sheng, Yuyan Zhang, Shunwang Li, Jing Zhang, Yuhao Zhang, Zheng-gen Jin, Zhuang Tian, Jiandong Jiang, Yanni Xu, Shuyi Si

**Affiliations:** 1 State Key Laboratory of Bioactive Substances and Functions of Natural Medicines, NHC Key Laboratory of Biotechnology for Antibiotics, National Center for New Microbial Drug Screening, Institute of Medicinal Biotechnology, Chinese Academy of Medical Sciences & Peking Union Medical College (CAMS&PUMC), No.1 Tiantan Xili, Beijing, 100050, China.; 2 Department of Clinical Pharmacy, Shanghai General Hospital, Shanghai Jiao Tong University School of Medicine, Shanghai, 200000, China.; 3 Pharmacy Department, Peking Union Medical College Hospital, PUMC & CAMS, Beijing, 100730, China.; 4 Department of Medicine, Aab Cardiovascular Research Institute, University of Rochester School of Medicine and Dentistry, 601 Elmwood Ave, Box CVRI, Rochester, NY 14642, USA.; 5 Cardiology Department, Peking Union Medical College Hospital, PUMC & CAMS, No.1 Shuaifuyuan, Beijing, 100730, China.

**Keywords:** leflunomide, teriflunomide, atherosclerosis, DHODH, AMPK

## Abstract

The probability of cardiovascular events has been reported lower in rheumatoid arthritis (RA) patients treated with leflunomide. However, the anti-atherosclerotic and cardiovascular protective effects and metabolism of leflunomide are not explored. In this study, we assessed the potential benefits of leflunomide on atherosclerosis and revealed the underlying mechanism. *ApoE^-/-^* mice were fed a western diet (WD) alone or supplemented with leflunomide (20 mg/kg, oral gavage, once per day) for 12 weeks. Samples of the aorta, heart, liver, serum, and macrophages were collected. We found that leflunomide significantly reduced lesion size in both *en-face* aortas and aortic root in WD-fed *ApoE^-/-^* mice. Leflunomide also obviously improved dyslipidemia, reduced hepatic lipid content, and improved disorders of glucose and lipid metabolism *in vivo*. RNA-Seq results showed that leflunomide effectively regulated the genes' expression involved in the lipid metabolism pathway. Importantly, leflunomide significantly increased the phosphorylation levels of AMPKα and acetyl-CoA carboxylase (ACC) *in vivo*. Furthermore, leflunomide and its active metabolite teriflunomide suppressed lipid accumulation in free fatty acid (FFA)-induced AML12 cells and improved endothelial dysfunction in palmitic acid (PA)-induced HUVECs through activating AMPK signaling and inhibiting dihydroorotate dehydrogenase (DHODH) signaling pathway. We present evidence that leflunomide and teriflunomide ameliorate atherosclerosis by regulating lipid metabolism and endothelial dysfunction. Our findings suggest a promising use of antirheumatic small-molecule drugs leflunomide and teriflunomide for the treatment of atherosclerosis and related cardiovascular diseases (CVDs).

## Introduction

Cardiovascular diseases (CVDs) are the leading cause of mortality and morbidity worldwide 1. Atherosclerosis is the most important pathological process leading to CVD such as ischemic heart disease and stroke 1. Atherosclerosis is characterized by plaque in the arterial wall with disordered lipid metabolism, vascular inflammation, and endothelial dysfunction. The multiple atherogenic risk factors can be categorized as traditional factors (such as dyslipidemia, diabetes mellitus, hypertension, and obesity) and non-traditional factors (such as inflammation and immunity); these factors are involved in the development of atherosclerosis and CVD 2-4. Abnormal high plasma low-density lipoprotein cholesterol (LDL-C) level is a well-established major risk factor for CVD 5, 6. Hypertriglyceridemia and hypercholesterolemia, the most common form of dyslipidemia 6, are associated with an increased risk of atherosclerotic CVD (ASCVD). At present, the management of dyslipidemias is the main treatment of atherosclerosis.

Adenosine monophosphate-activated protein kinase (AMPK) is a cellular energy sensor that regulates multiple physiological processes including lipid and glucose metabolism and inflammation to maintain energy homeostasis. Overwhelming evidence indicates that pharmacological AMPK activation (polyphenol S17834, A-769662, AICAR, metformin, and IMM-H007) attenuates atherosclerosis development in diet-induced apolipoprotein E deficient (*ApoE^-/-^*) or LDL receptor-deficient (*LDLR^-/-^*) mice, which can be explained by their beneficial effects on hepatic lipid accumulation, hyperlipidemia, insulin resistance, and autophagy. AMPK suppresses cholesterol and fatty acid synthesis and reduces lipid storage through phosphorylation of HMG-CoA reductase (HMGR) and acetyl-CoA carboxylase (ACC)^7^-^11^. AMPK can also inhibit the activity of sterol regulatory element binding protein (SREBP), a key lipogenic transcription factor, may offer therapeutic strategies to combat insulin resistance, dyslipidemia, and atherosclerosis 12. In addition, endothelial AMPK is an important factor in tissues exposed to ischemic stress 13. Therefore, therapeutic targeting activation of AMPK is a potential strategy for treating atherosclerosis.

Chronic systemic inflammation, caused by immune dysregulation, is a key factor in the pathological process of atherosclerosis. CVD is the most common cause of mortality among patients with rheumatoid arthritis (RA), which is a chronic systemic inflammatory autoimmune disorder 14. The CVD risk among RA patients is 2-fold greater than that of the general population because of the high prevalence of traditional and non-traditional risk factors, which lead to premature atherosclerosis, endothelial dysfunction, and atherosclerogenesis 15. Leflunomide is widely used in the treatment of RA. Leflunomide and its active metabolite teriflunomide play an immunoregulatory role by inhibiting the activity of dihydroorotate dehydrogenase (DHODH), which is the pyrimidone biosynthesis rate-limiting enzyme located at the inner mitochondrial membrane 16, 17. Previous studies indicated that leflunomide significantly reduces the incidence of cardiovascular events, such as acute myocardial infarction, in RA patients 18, 19. An increasing number of studies have reported the anti-inflammatory effect of leflunomide 20-23. Furthermore, leflunomide can suppress the migration of peripheral blood mononuclear cells 24, which is involved in the initial stage of formation of atherosclerotic plaques 2, 3, 24. Although the available evidence suggests that leflunomide might have anti-atherosclerosis effects, direct evidence of the anti-atherosclerosis and cardiovascular benefits of leflunomide and the possible underlying mechanisms remain to be elucidated.

In this study, we first explored the potential benefits of leflunomide on atherosclerosis and revealed the underlying mechanism in *ApoE^-/-^* mice. We also assessed the *in vitro* anti-atherosclerotic and cardiovascular protective effects of leflunomide and teriflunomide, as well as assessed the underlying mechanisms. Our data demonstrated that leflunomide and teriflunomide have positive regulatory effects on lipid metabolism and vascular protective effects through activating AMPK and these effects could be blocked when DHODH was overexpressed. Taken together, our study suggests that antirheumatic small-molecule drugs leflunomide and its active metabolite teriflunomide have a promising use for the treatment of atherosclerosis and related CVDs.

## Methods

### Materials

Leflunomide (CAS: 75706-12-6, Cat No. L0250) and teriflunomide (CAS: 108605-62-5, Cat No. T3287) were purchased from TCI Chemicals (Shanghai, China). Palmitic acid (PA, CAS: 57-10-3, Cat No. P0500) and oleic acid (OA, CAS: 112-80-1, Cat No. O1008) were purchased from Sigma-Aldrich (St. Louis, MO, USA).

### Cell culture

Alpha mouse liver 12 (AML12) cells were purchased from ATCC (Manassas, VA, USA) and maintained in Dulbecco's modification of Eagle's medium Dulbecco (DMEM, Thermo Fisher Scientific, Waltham, MA, USA) containing 10% fetal bovine serum (FBS) (Thermo Fisher Scientific). Human umbilical vein endothelial cells (HUVECs) (ATCC) were cultured in an endothelial cell growth medium (PromoCell, Heidelberg, Germany). HUVECs between P3 and P6 were used for all experiments. All cells were cultured at 37°C in a humidified atmosphere containing 5% CO_2_.

### Animals and treatment

All animal care and experimental protocols in this study conformed to the Guide for the Care and Use of Laboratory Animals by the Institute of Medicinal Biotechnology, Chinese Academy of Medical Sciences and Peking Union Medical College (Beijing, China, Approval Number: IMB-20210514D3). Animal studies are reported in compliance with the ARRIVE guidelines 25.

Eight-week-old male *ApoE^-/-^* mice were purchased from Beijing Vital River Laboratory Animal Technology, Co., Ltd. (Beijing, China). The atherosclerotic model was induced by a western diet (WD, Cat No. TP26300, 21% fat, 0.2% cholesterol, 49.1% carbohydrate, and 19.8% protein; Trophic Animal Feed High-tech, Co., Ltd., Nantong, China) as we previously described 26. Fifty-four mice were randomly divided into three groups (n = 18 for each group) and received the following treatments: normal chow diet (ND) group, mice were fed ND (Cat No. Co60, SPF; Beijing Biotechnology, Co., Ltd.) and intragastrically administered with 0.5% carboxymethylcellulose-Na (CMC-Na); WD group mice were fed WD and were intragastrically administered with 0.5% CMC-Na; leflunomide group, mice were fed WD and intragastrically administered with leflunomide (20 mg/kg body weight, dissolved in 0.5% CMC-Na). Leflunomide or vehicle (0.5% CMC-Na) was given to *ApoE^-/-^* mice once per day for 12 weeks. At the end of treatment, mice were fasted overnight and anesthetized with isoflurane, and killed via cervical dislocation. Plasma samples, aortas, heart, liver, and other organs were excised and fixed in 4% paraformaldehyde or stored at -80°C for further analysis.

### Lesion size assessment in *ApoE^-/-^* mice

The atherosclerotic lesion was analyzed as previously described 26. The ORO staining images of aortas were captured using a digital camera under the microscope (Leica, Wetzlar, Germany, DFC450C). The frozen cross-sections of aortic sinus tissues (8 μm) were prepared for ORO and hematoxylin & eosin (H&E) staining to measure the atherosclerotic lesion size. The stained sections were scanned using PANNORAMIC MIDI II (3DHISTECH Ltd., Budapest, Hungary). The lesions on the *en-face* aortas and stained aortic sinus cross sections were analyzed using the National Institutes of Health ImageJ software.

### Plasma lipid and biochemical indexes and inflammatory cytokines measurements

Commercially available kits (Biosino Biotechnology and Science, Beijing, China) were used to determine the plasma biochemical indexes, including total cholesterol (TC), triglyceride (TG), glucose, alanine aminotransferase (ALT), and aspartate aminotransferase (AST). Plasma insulin (Cat No. SBJ-M0583), glycosylated hemoglobin A1c (GHbA1c) (Cat No. SBJ-M0563), interleukin-1*β* (IL-1*β*) (Cat No. SBJ-M0583), tumor necrosis factor-alpha (TNF*α*) (Cat No. SBJ-M0030), and interleukin-6 (IL-6) (Cat No. SBJ-M0657) levels were determined using enzyme-linked immunosorbent assay kit (Sbjbio, Nanjing, China).

Fast protein liquid chromatography (FPLC) assay was performed to determine the plasma lipoprotein profiles as previously described 27. Briefly, 150 μl of plasma per group were loaded onto a Superose Increase 6 10/300 GL column (GE Healthcare Europe GmbH, Munich, Germany) and separated on ÄKTA explorer analysis system (GE, Healthcare Europe GmbH) eluted at a speed of 0.2 ml buffer per min. The TC concentration in the fractions was measured in each fraction using the assay kit mentioned above.

### Hepatic lipid content

Lipids from livers were homogenized at 4°C using GeneReady Ultimate (Hangzhou LifeReal Biotechnology Co., Ltd, Zhejiang, China). After centrifugation, the supernatants were collected, and TC and TG concentrations were measured as described above. The protein contention was determined by the bicinchoninic acid (BCA) protein assay kit (Thermo Fisher Scientific, Waltham, MA, USA, Cat No. 23225). Liver TC and TG concentrations were expressed as μmol per gram protein (μmol /g protein).

### Histological analysis of liver

Livers from mice were fixed in 4% paraformaldehyde overnight and stored in 20% sucrose at 4°C. Liver paraffin sections were stained with H&E, and the frozen cross sections were stained with ORO. The stained sections were scanned using PANNORAMIC MIDI II (3DHISTECH Ltd., Budapest, Hungary). The ORO staining areas of liver cross sections were analyzed using the ImageJ software and expressed as percentages of the whole area.

### Oral glucose tolerance test (OGTT)

Seven days before the end of administration, eight mice from each group were fasted for 6 h. Immediately after the 0-min sample was taken, each mouse was administered glucose solution (2 g/kg body weight) by gavage. The blood glucose levels were measured from the tip of the tail at time points 15, 30, 60, 90, and 120 min using a glucometer (Roche, Basel, Switzerland). The area under the curve (AUC) was calculated.

### Insulin tolerance test (ITT)

Seven days before the end of the administration, another cohort of eight mice from each group was fasted for 4 h for the ITT. Immediately after the 0-min sample was taken, each mouse was intraperitoneally injected with insulin solution (0.75 IU/kg body weight). The blood glucose levels were measured from the tip of the tail at the time points 15, 30, 60, 90, and 120 min after injection. The AUC was calculated.

### Immunofluorescence Assay

An immunofluorescence staining assay was performed on treated AML12 cells. The cells were stained with the primary antibodies against sterol regulatory element binding protein 1 (SREBP1) at 4°C overnight and then incubated with the secondary antibodies (goat anti-mouse Alexa Fluor 488®, Thermo Fisher Scientific, Cat No. A-11001) for 30 minutes at 37°C. The cell nuclei were counterstained with DAPI (4',6-diamidino-2-phenylindole) (Thermo Fisher Scientific, Cat No. 62248) for 10 minutes. The images were captured using the High Content Analysis System (Operetta CLS™, PerkinElmer, Fremont, CA, USA).

### RNA sequencing (RNA-Seq) analysis

RNA-Seq analysis of liver tissues or aortas from WD-fed *ApoE^-/-^* mice was performed by BMK company (Beijing, China) and analyzed using BMKCloud (www.biocloud.net). The genes with ≥ 1.5-fold change and *P* value < 0.01 were considered to have a significant difference and were defined as differentially expressed genes (DGEs). A Kyoto Encyclopedia of Genes and Genomes (KEGG) and gene set enrichment analysis (GSEA) pathway enrichment analysis were carried out to find important signaling pathways.

### Effect of leflunomide and teriflunomide on lipid accumulation in AML12 cells

AML12 cells were treated with free fatty acid (FFA, 500 μM totally; PA: OA = 1:2) and different concentrations of leflunomide and teriflunomide for 18h simultaneously in 96-well or 6-well plates. Then the cells were stained by Nile red (Sigma-Aldrich, Cat No. 19123) and the images were captured using a Leica microscope. The relevant proteins were detected by Western blot or immunofluorescence staining assay.

### Effect of leflunomide and teriflunomide in HUVECs

HUVECs were treated with PA (500 μM) and different concentrations of leflunomide and teriflunomide for 18h simultaneously. Nitric oxide (NO) production was determined in the following experiment. The relevant proteins were detected by Western blot.

### SiRNA knockdown assay

HUVECs or AML12 cells were transfected with control siRNA (si-control) or AMPKα siRNA (si-AMPKα) (60 nM, Santa Cruz Biotechnology) using Lipofectamine RNAi^MAX^ (Thermo Fisher Scientific, Cat No. 13778100). Then, the cells were treated with leflunomide, teriflunomide, or vehicle with or without FFA stimulation at the indicated concentration and time.

### DHODH overexpression assay

The control plasmid pCDNA3.1(+) was obtained from Thermo Fisher Scientific (Waltham, MA, USA). To construct the mouse or human DHODH overexpression plasmid, the gene segment of mouse DHODH (Gene bank ID: 56749) or human DHODH (Gene bank ID: 1723) was cloned into pCDNA3.1(+) (named pCDNA3.1-mDHODH or pCDNA3.1-hDHODH), respectively by GenScript Biotech Corporation (Nanjing, China). The plasmid pCDNA3.1-DHODH (2 μg/ml) or control empty vector pCDNA3.1(+) were transfected into HUVECs or AML12 cells with lipofectamine 2000 for 24 h. Then the cells are treated with leflunomide, teriflunomide, or vehicle with or without FFA or PA stimulation at the indicated concentration and time.

### NO production measurement

HUVECs were treated as above needed. Then the cells were loaded to DAF-FM DA (Diaminofluorescein-FM diacetate) (5 μM, Beijing Solarbio Science & Technology), a commercial NO-sensitive fluorescent probe, at 37°C for 30 min. The production level of intracellular NO was detected based on the fluorescence intensity of DAF-FM DA at 495 nm after excitation at 515 nm using an Envision microplate reader (PerkinElmer, Fremont, CA, USA).

### Western blot analysis

Western blot analysis was performed as described 26. The relative protein levels of target genes were normalized to *β*-actin in the corresponding sample to reduce variance. The antibodies were shown in the Supplementary data. All values were normalized to the mean value of the experimental control group. The data were expressed as folds of the control group's mean value.

### Quantitative real-time PCR (qRT-PCR)

Total RNA was extracted using a QIAGEN RNeasy Mini kit (Qiagen, Hilden, Germany). The RNA was converted into complementary DNA (cDNA) using the TransScript One-Step gDNA Removal and cDNA synthesis kit (TransGen Biotech, Beijing, China). Then, qRT-PCR was performed using corresponding primers and FastStart Universal SYBR Green Master (Roche) on FTC-3000 Real-Time Quantitative Thermal Cycler (Funglyn Biotech Inc., Richmond Hill, Canada). The used primers are shown in **Supplementary Table 1**.

### Statistical analysis

The data analysis was performed in a blinded manner. The data were presented as mean ± SEM. When comparing two groups, an unpaired or paired two-tailed Student's *t*-test was performed after a normality test. When comparing three or more groups, one-way ANOVA followed by the Bonferroni post hoc test was used. All analyses were performed by GraphPad Prism 8 (GraphPad Software, CA, USA). *P* value < 0.05 was considered significant.

## Results

### Leflunomide reduces plasma lipid and glucose levels in WD-fed *ApoE^-/-^* mice

**Figure 1:**
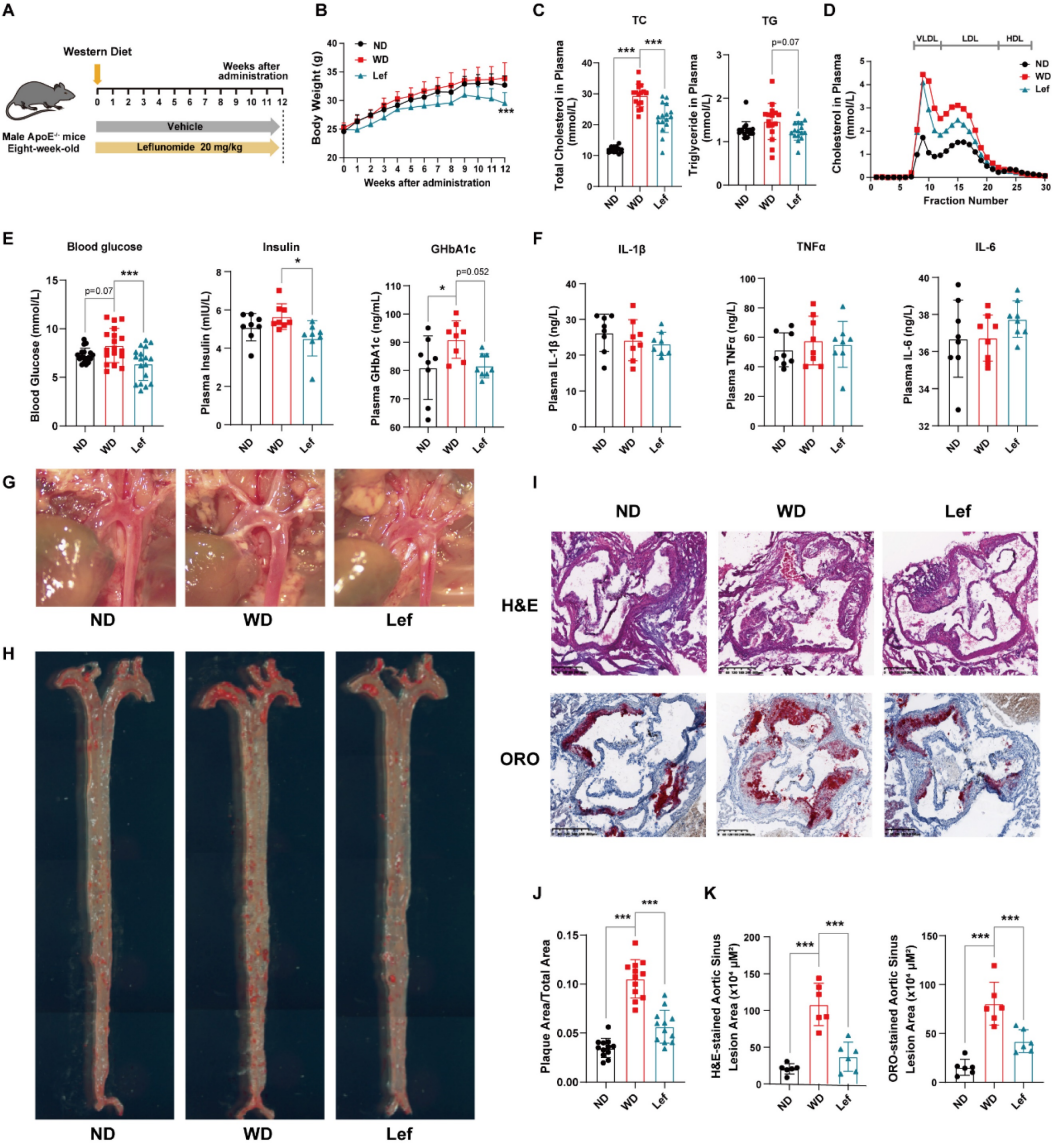
** Leflunomide exerts an anti-atherosclerotic effect in WD-fed *ApoE^-/-^* mice. (A)** Schematic diagram of *ApoE^-/-^* mice treatment. **(B)** The body weight of *ApoE^-/-^* mice we investigated was monitored once a week for 12 weeks. n = 18 per group. **(C)** The TC and TG levels in the plasma of *ApoE^-/-^* mice in ND, WD, and leflunomide treatment (named Lef group) group. n = 18 per group.** (D)** Lipoproteins in the plasma of *ApoE^-/-^* mice in ND, WD, and Lef groups were separated by FPLC, and the TC level in the fractions was determined. **(E)** The plasma blood glucose (n = 18 per group), insulin (n = 8 per group), and GHbA1c (n = 8 per group) levels. **(F)** The IL-1*β*, TNF*α,* and IL-6 levels in the plasma. n = 8 per group.** (G)** Respective captured digital images of aortas. **(H)** Respective images of *en-face* aortas stained by ORO. **(J)** The *en-face* aorta lesions area was quantitatively analyzed with Image J software. n = 12 per group.** (I, K)** Respective images of H&E and ORO-stained aortic sinus cross sections of each group **(I)**, and all stained sections were analyzed and quantified with Image J software **(K)**. Scale bar = 500 μm, n = 6 per group. One-way ANOVA analysis was performed: *P < 0.05, ** P < 0.01, *** P < 0.001.

The formation of atherosclerotic lesions is a lipid-driven and glucose-accelerated pathological process 28. To test whether leflunomide has a direct anti-atherosclerotic effect, *ApoE^-/-^* mice were fed a WD and treated with leflunomide or vehicle for 12 weeks (**Figure 1A**). As shown in **Figure 1B**, leflunomide treatment had significant body weight loss compared to those in the WD group at the endpoint, while there was no significant change between ND and WD groups. As illustrated in **Figure 1C**, plasma TC and TG levels of mice fed with WD for 12 weeks were significantly higher than those in the ND group mice. Leflunomide treatment markedly decreased the plasma TC levels (28.34 ± 4.02% vs. 21.07 ± 6.26%) and TG levels (1.64 ± 0.32% vs. 1.33 ± 0.13%) compared with those in the WD group mice. FPLC assay showed that the WD group showed a significant increase in cholesterol levels in plasma very low-density lipoprotein (VLDL) and LDL particle levels in the lipoprotein profile compared to the ND group, while leflunomide treatment led to significantly reduced VLDL-C and LDL-C levels compared to the WD group mice (**Figure 1D**). In addition, as presented in **Figure 1E**, the fasting plasma glucose, insulin, and GHbA1c levels significantly increased in WD mice compared with the ND group; however, the fasting plasma glucose, insulin, and GHbA1c levels were significantly decreased in the leflunomide-treated group compared to WD group. To sum up, these data indicate that leflunomide treatment has clear beneficial effects on lipid and glucose metabolism.

Chronic inflammatory response is another important pathological characteristic of atherosclerosis, we analyzed the expression levels of inflammatory cytokines in plasma and peritoneal macrophages from *ApoE^-/-^* mice, respectively. As shown in **Figure 1F**, there were no significant differences in cytokines including IL-1*β*, TNF*α,* and IL-6 between the groups. However, the mRNA level of *Il-1β* and *Tnfα* in the peritoneal macrophages of the leflunomide-treated mice was significantly lower compared to the WD group **(Supplementary Figure 1A-C)**.

Considering the important role of reverse cholesterol reversal (RCT) process in macrophage foaming and atherosclerosis pathology 29, we examined the genes related to RCT including ATP binding cassette transporter A1 (ABCA1), ATP binding cassette transporter G1 (ABCG1), scavenger receptor class B type I (SR-BI), scavenger receptor A (SR-A) and scavenger receptor CD36 in mouse peritoneal macrophages. As shown in **Supplementary Figure 1D**, we did not observe significant differences that occurred after leflunomide treatment among these genes (*Abca1, Abcg1, Sr-b1, Cd36*, and *Sr-a*), suggesting that leflunomide treatment might not affect the RCT process* in vivo*.

### Leflunomide treatment attenuates atherosclerosis in WD-fed *ApoE^-/-^* mice

As shown in **Figure 1G**, leflunomide significantly reduced plaque area in the aortic arch compared to that in the WD group. ORO staining results of *en-face* aorta showed that WD group mice had significantly increased atherosclerotic plaque areas than the ND group (10.54 ± 1.95% vs. 3.49 ± 0.79%); however, leflunomide-treated group mice exhibited significantly smaller lesion area comparing the WD group (5.65 ± 1.67% vs. 10.54 ± 1.95%) (**Figure 1H&J**). H&E and ORO staining of the aortic root cross sections showed that the lesion areas of WD group mice were significantly greater than those of the ND group (80.40 ± 21.84 vs. 15.30 ± 8.22 × 10^4^ μm^2^ for H&E staining; 115.90 ± 24.07 vs. 20.80 ± 7.82 × 10^4^ μm^2^ for ORO staining; **Figure 1I&K**); however, the lesion areas of leflunomide-treated group mice were significantly less comparing the WD group (41.99 ± 11.68 vs. 80.40 ± 21.84 × 10^4^ μm^2^ for H&E staining; 41.82 ± 18.02 vs. 115.90 ± 24.07 × 10^4^ μm^2^ for ORO staining; **Figure 1I&K**). These results suggest that leflunomide treatment has a strong anti-atherosclerotic effect in WD-fed *ApoE^-/-^* mice.

### Leflunomide treatment improves liver lipid and glucose metabolism in WD-fed *ApoE^-/-^* mice

**Figure 2:**
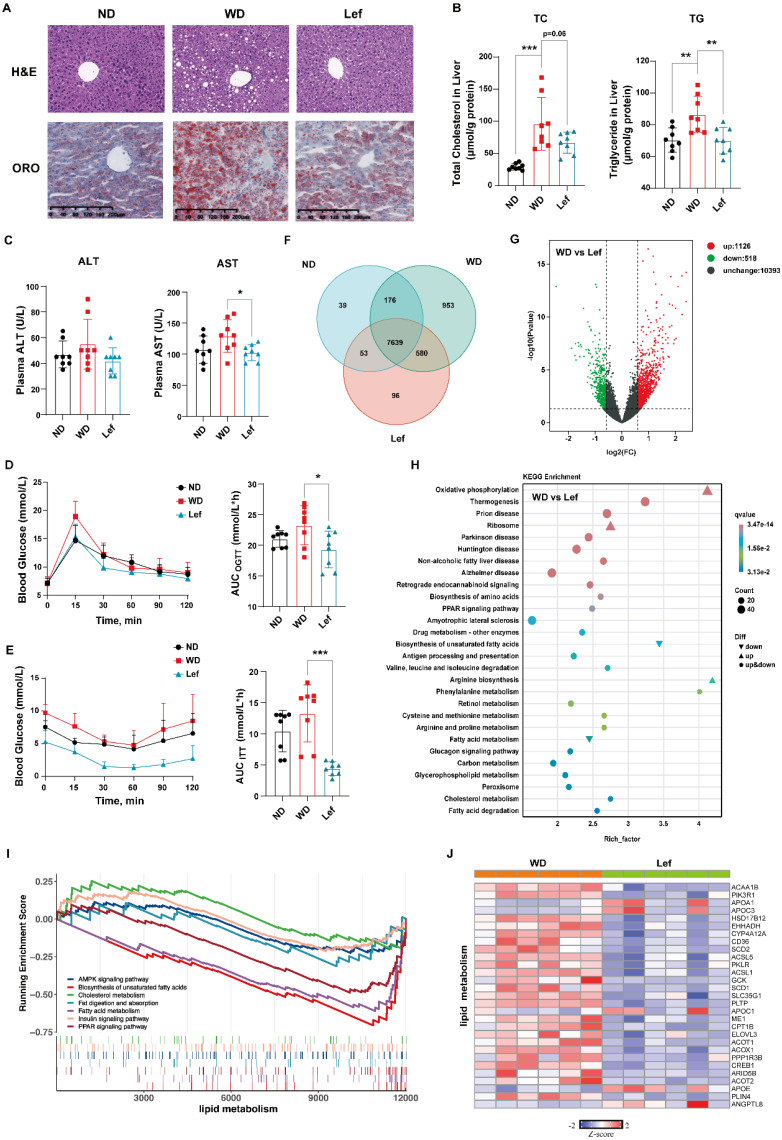
** Leflunomide treatment improved lipid and glucose metabolism in WD-fed *ApoE^-/-^* mice. (A)** The respective images of the H&E and ORO-stained section of the liver in the ND, WD, and leflunomide treatment (named Lef group) group *ApoE^-/-^* mice. Scale bar = 200 μm, n = 6 per group. **(B)** The TC and TG levels in the liver of the ND, WD, and Lef groups. n = 8 per group. **(C)** The plasma ALT and AST levels. n = 16 per group. **(D-E)** The blood glucose levels at a certain time point were detected during the OGTT and ITT and the areas under the curve (AUC) were calculated respectively. **(F)** Venn diagram based on RNA-Seq results from the livers of ND, WD, and Lef groups. n = 6 per group. **(G)** Volcano map of differentially expressed genes (DGEs) (fold change ≥ 1.5 and *P* < 0.01, WD vs Lef) in RNA-Seq results from the livers of WD and Lef groups. n = 6 per group.** (H)** KEGG enrichment analysis DGEs between WD and Lef group. n = 6 per group. **(I)** GSEA enrichment results show the pathways related to lipid metabolism in the RNA-Seq dataset. n = 6 per group. **(J)** Heatmap of lipid metabolism-related DGEs profiles based on the RNA-seq data set. *P* < 0.01, n = 6 per group. One-way ANOVA analysis was performed: *P < 0.05, ** P < 0.01, *** P < 0.001.

Since the excessive lipid deposits lead to hepatocyte damage and steatosis 30, we evaluated the effects of leflunomide on lipid metabolism in livers of *ApoE^-/-^* mice. H&E staining results showed that there was obvious macrovesicular steatosis in the livers of WD-fed *ApoE^-/-^* mice compared to ND mice, while leflunomide treatment significantly improved hepatic steatosis and protected the hepatocytes compared to the WD group (**Figure 2A**). The ORO staining results of mice liver sections showed that leflunomide effectively reduced hepatic lipid accumulation (**Figure 2A**). TC and TG levels in the livers of the WD group mice were significantly higher than those in the ND group (**Figure 2B**). Leflunomide treatment substantially decreased hepatic TC and TG contents compared to the WD group (**Figure 2B**). In addition, the plasma AST but not ALT levels were significantly decreased after the administration of leflunomide compared with the WD group (**Figure 2C**). These results suggest that leflunomide could alleviate the liver lipid accumulation and liver damage.

Abnormal glucose metabolism is an independent risk factor for CVD, and insulin resistance often coexists with common proatherogenic disorders 31. *ApoE^-/-^* mice are prone to insulin resistance when fed with WD 32. Hence, OGTT and ITT were performed to determine whether leflunomide can affect glucose metabolism and insulin sensitivity in *ApoE^-/-^* mice. As shown in **Figure 2D-E**, leflunomide treatment markedly decreased the AUC of OGTT and ITT compared to the WD mice, which indicates that leflunomide significantly improved glucose tolerance and insulin sensitivity of leflunomide-treated mice.

Collectively, these results suggest that the daily administration of leflunomide improves lipid and glucose metabolism, key CVD risk factors, in WD-fed *ApoE^-/-^* mice.

### Transcriptomic profile in leflunomide-treated livers of WD-fed *ApoE^-/-^* mice from RNA-Seq

To comprehensively explore the mechanism of the effect of leflunomide on atherosclerosis, RNA-Seq analysis of liver tissues was performed. The Venn diagram revealed the overlapping genes in the three groups (**Figure 2F**). The volcano plot diagram showed the numbers of DGEs were 1128 up-regulated and 518 down-regulated (WD vs. leflunomide group), respectively (**Figure 2G**). KEGG enrichment analysis revealed that leflunomide mainly regulated DEGs within pathways related to lipid metabolism (such as oxidative phosphorylation, non-alcoholic fatty liver disease, biosynthesis of amino acids, peroxisome proliferators-activated receptors (PPAR) signaling pathway, fatty acid metabolism, cholesterol metabolism, and fatty acid degradation) (**Figure 2H**). GSEA pathway enrichment analysis results showed that leflunomide regulated lipid metabolism-related signaling pathways (such as AMPK, biosynthesis of unsaturated fatty acids, cholesterol metabolism, fat digestion and absorption, fatty acid metabolism, insulin, and PPAR) (**Figure 2I**). A heatmap based on the KEGG and GSEA results also revealed that genes (such as *Cd36*, *Scd1*, *Scd2*, *Apoc3*, *Acot1*, and *Elovl3*) related to lipid metabolism pathways were significantly down-regulated by leflunomide treatment compared with the WD group. What's more, GSEA analysis results revealed that pathways and genes related to inflammation (such as mitogen-activated protein kinase (MAPK) and cytokine-cytokine receptor interaction pathways) were also significantly down-regulated after leflunomide treatment (**Supplementary Figure 2A-B**). Overall, these data demonstrate that leflunomide exerts potent anti-atherosclerosis effects through regulating lipid metabolism.

### Leflunomide activates AMPK pathway in WD-fed *ApoE^-/-^* mice

**Figure 3:**
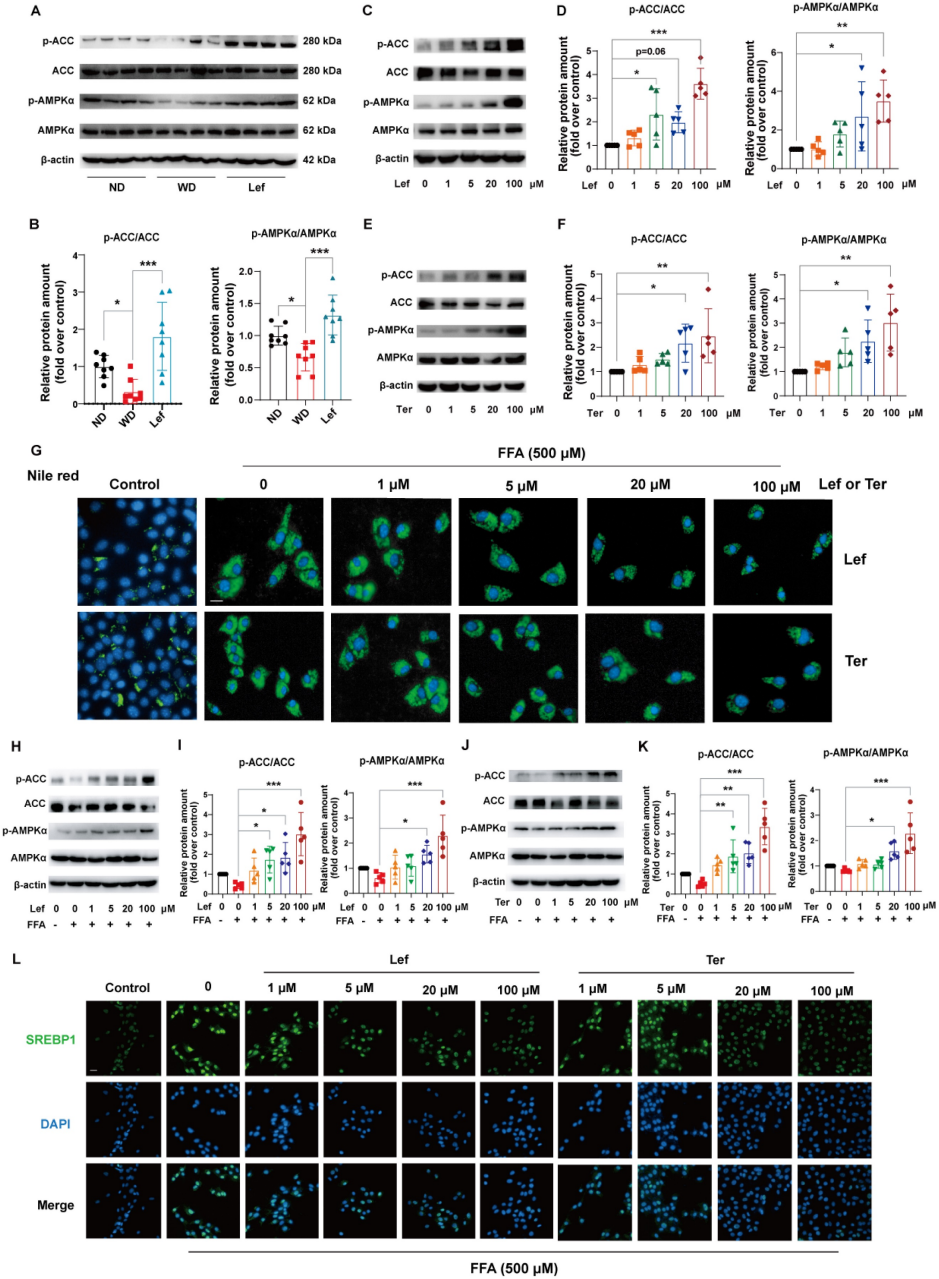
** Leflunomide and Teriflunomide activate the AMPK pathway and alleviate lipid accumulation in FFA-stimulated AML12 cells. (A-B)** Western blot analysis of the p-AMPKα, AMPKα, p-ACC, and ACC in livers of *ApoE^-/-^* mice in ND, WD, and leflunomide treatment (named Lef) group. Representative immunoblots are shown **(A)** and the images were quantitatively analyzed with Image J software **(B)**, and then the ratio of p-AMPKα/AMPKα and p-ACC/ACC was calculated **(B)**. n = 8 per group. **(C-F)** AML12 cells were treated with leflunomide (0, 1, 5, 20, 100 μM) **(C-D)** or teriflunomide (0, 1, 5, 20, 100 μM) **(E-F)** for 30 min (0, 1, 5, 20, 100 μM). The protein levels of p-AMPKα, AMPKα, p-ACC, and ACC were detected and the ratio of p-AMPKα/AMPKα and p-ACC/ACC was quantitatively analyzed. n = 5. **(G-L)** AML12 cells were treated with or without FFA (500 μM, PA: OA = 1:2) and leflunomide (0, 1, 5, 20, 100 μM) or teriflunomide (0, 1, 5, 20, 100 μM) simultaneously for 18 h. **(G)** The respective images of Nile red staining. Scale bar = 20 μm. n = 5. **(H-K)** Representative immunoblot images quantitative analysis of the ratio of p-AMPKα/AMPKα and p-ACC/ACC. n = 5.** (L)** Representative immunofluorescent images of SREBP1 (green) and the cell nucleus stained with DAPI (blue). Scale bar = 20 μm. n = 5. Leflunomide is abbreviated as Lef, and teriflunomide is abbreviated as Ter in all *in vitro* assays. One-way ANOVA analysis was performed: *P < 0.05, ** P < 0.01, *** P < 0.001.

Phosphor-acetyl CoA carboxylase (p-ACC) and ACC were the inactivated and activated forms of the de novo fatty acid synthesis rate-limiting enzymes, respectively 33. AMPK may lower the liver lipid synthesis rate through phosphorylation of ACC to cause their inactivation 34. GSEA pathway enrichment analysis results showed that leflunomide regulated the AMPK signaling pathway (**Figure 2I**). We examined the protein levels of phospho-AMPKα (p-AMPKα), AMPKα, p-ACC, ACC, and peroxisome proliferative activated receptor gamma coactivator 1 alpha (PGC1α), a target gene of the AMPK signaling pathway, in the livers of the three groups. As shown in **Figure 3A-B & Supplementary Figure 3A-B**, leflunomide treatment significantly increased p-AMPKα and p-ACC expression, the ratio of p-AMPKα/AMPKα, the p-ACC/ACC ratio, and PGC1α compared to the WD group, indicating that leflunomide indeed activated the AMPK signaling pathway* in vivo*. However, leflunomide had no significant effect on the protein levels of SREBP1, SREBP2, HMGCR, LXRα, and FASN (**Supplemental Figure 4**). These data demonstrate that leflunomide activates the AMPK signaling pathway and may explain its *in vivo* lipid metabolism regulation ability.

### Leflunomide and teriflunomide activate the AMPK pathway and alleviate lipid accumulation *in vitro*

Teriflunomide is the metabolite of leflunomide that is the major contributor to immunosuppression through inhibiting the synthesis of pyrimidine *in vivo* 17. We further investigated the effects of leflunomide and teriflunomide on lipid accumulation and the AMPK signaling pathway in mouse liver AML12 cells. Our data showed that leflunomide (**Figure 3C-D**) and teriflunomide (**Figure 3E-F**) led to a significant increase in the p-AMPKα/AMPKα and p-ACC/ACC ratio in AML12 cells treated with the two compounds for 30 min, which is consistent with the findings in *ApoE^-/-^* mice. Furthermore, Nile red staining showed that both leflunomide and teriflunomide effectively reduced the cellular lipid droplet accumulation in FFA-treated AML12 cells with a dose-dependent effect (**Figure 3G**). Western blot results showed that leflunomide (**Figure 3H-I**) and teriflunomide (**Figure 3J-K**) significantly increased the p-AMPKα/AMPKα and p-ACC/ACC ratio of protein level in FFA-stimulated AML12 cells, and the similar results were also observed in the human hepatocyte cell line L02 (**Supplementary Figure 5**). Immunofluorescent staining results showed that leflunomide and teriflunomide reduced the nucleus SREBP1 expression, an important lipid homeostasis regulatory gene downstream of the AMPK signaling pathway, in FFA-stimulated AML12 cells (**Figures 3L**). These results suggest that leflunomide and teriflunomide regulate lipid metabolism by activating the AMPK pathway* in vitro*.

### Leflunomide and teriflunomide alleviate lipid metabolic profile via DHODH/AMPK pathway in AML12 cells

**Figure 4:**
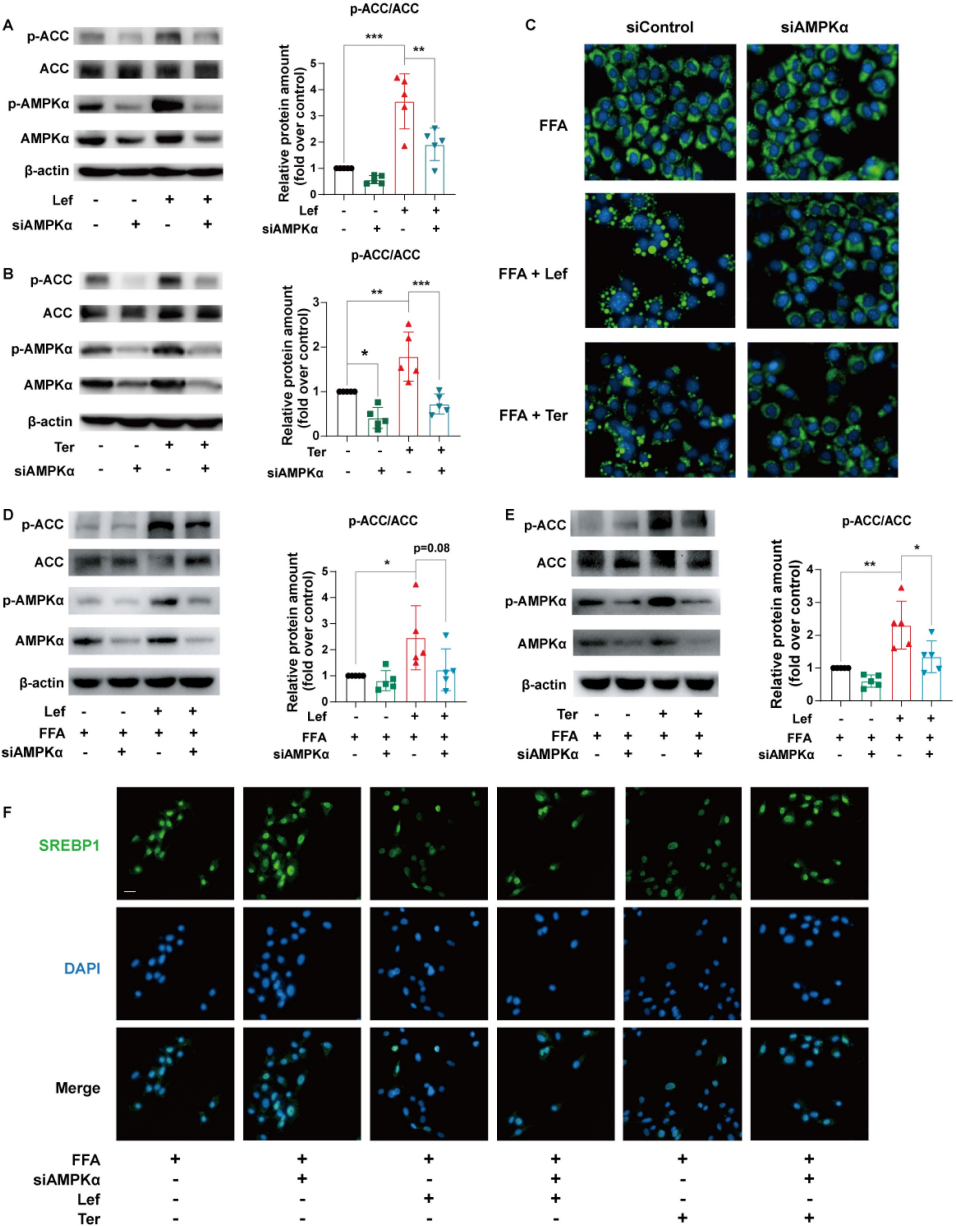
** Leflunomide alleviates lipid accumulation in FFA-stimulated AML12 cells via the AMPK pathway. (A-B)** AML12 cells were transfected with control siRNA (si-control) or AMPKα siRNA (si-AMPKα) for 24 h, and then treated with or without leflunomide (100 μM) or teriflunomide (100 μM) for 30 min. The representative immunoblots of p-AMPKα, AMPKα, p-ACC, and ACC were shown and the p-ACC/ACC ratio was calculated. n = 5. **(C-E)** AML12 cells were first transfected with si-control or si-AMPKα for 24 h and then were treated with FFA and leflunomide (100 μM) or teriflunomide (100 μM) simultaneously for 18 h. **(C)** The Nile red stain was performed and the respective images were shown. Scale bar = 20 μm. **(D-E)** The representative immunoblots of p-AMPKα, AMPKα, p-ACC, and ACC were shown, and the p-ACC/ACC ratio was calculated. n = 5.** (F)** Representative immunofluorescent images of SREBP1 (green) and the cell nucleus stained with DAPI (blue). n = 5. One-way ANOVA analysis was performed: *P < 0.05, ** P < 0.01, *** P < 0.001.

To explore the role of the AMPK signaling pathway in the improvement of lipid metabolism by leflunomide and teriflunomide, AMPKα, the critical residue for activation of the AMPK signaling pathway, was knocked down using specific siRNA in AML12 cells. Our results showed that the increased effects of leflunomide and teriflunomide on p-AMPKα and p-ACC were significantly diminished when AMPKα was knockdown (**Figure 4A-B**). AMPKα deficiency significantly reversed the inhibitory activity of leflunomide and teriflunomide on lipid accumulation in FFA-treated AML12 cells (**Figure 4C**), and the effects of elevated protein p-ACC/ACC ratio of leflunomide (*P* = 0.08) and teriflunomide (*P* < 0.05) were also reversed when AMPKα was knockdown (**Figures 4D-E**). Meanwhile, the modulatory effects of leflunomide and teriflunomide on SREBP1 were substantially diminished when AMPKα was absent in FFA-stimulated AML12 cells (**Figure 4F**).

**Figure 5:**
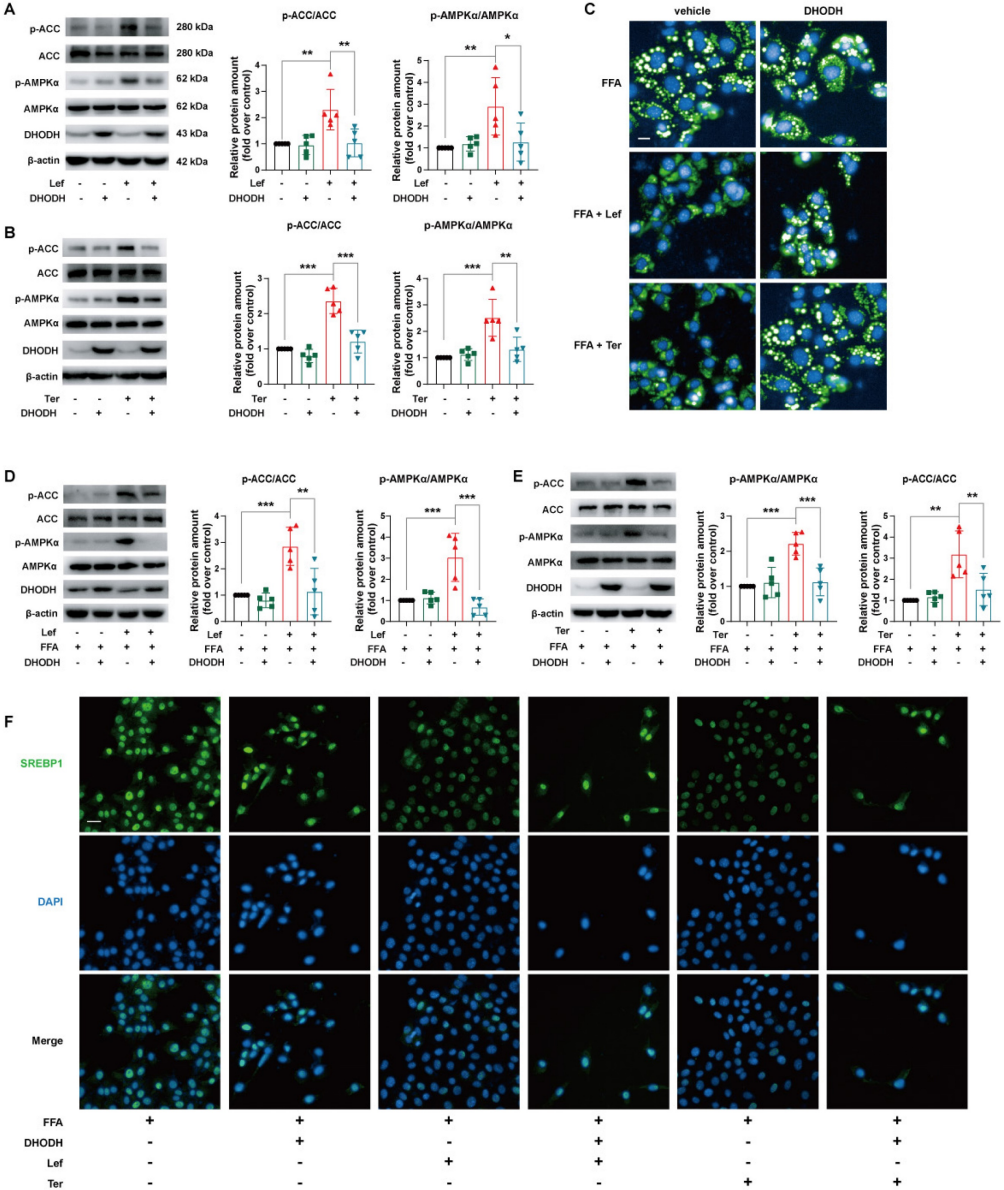
** The effects of leflunomide and teriflunomide on lipid accumulation and AMPK activation in AML12 cells were mediated by inhibiting DHODH. (A-B)** The AML12 cells were first transfected with empty vector pCDNA3.1 or plasmid pCDNA3.1-mDHODH for 24h, and then cells were treated with leflunomide (100 μM) or teriflunomide (100 μM) for 30 min.** (C-F)** After transfected with empty vector pCDNA3.1 or plasmid pCDNA3.1-mDHODH for 24 h, the AML12 cells were treated with FFA (500 μM) and leflunomide (100 μM) or teriflunomide (100 μM) simultaneously for 18 h. **(A-B & D-E)** The representative immunoblots of p-AMPKα, AMPKα, p-ACC, and ACC were shown and the ratio of p-ACC/ACC and p-AMPKα/AMPKα were analyzed and calculated. n = 5. **(C)** Nile red staining was performed and the respective images were shown. Scale bar = 20 μm. **(F)** The immunofluorescent staining was performed with specific SREBP1 antibody (green) and DAPI (blue) for cell nucleus staining. One-way ANOVA analysis was performed: *P < 0.05, ** P < 0.01, *** P < 0.001.

DHODH is a recognized target of leflunomide and teriflunomide 35, but there is no direct evidence that DHODH can be involved in the regulation of energy metabolism by the AMPK signaling pathway. The protein expression level of DHODH in the liver of *ApoE^-/-^* mice given leflunomide was significantly reduced compared with that of the WD group (**Supplementary Figure 6**). We then examined whether the regulating lipid metabolism ability of leflunomide and teriflunomide is associated with DHODH. Interestingly, the increased p-AMPKα and p-ACC protein expression activity of leflunomide and teriflunomide was significantly lessened when DHODH was overexpressed in AML12 cells (**Figure 5A-B**). The inhibitory effect on lipid droplet accumulation (**Figure 5C**), the increased p-AMPKα and p-ACC protein expression activity (**Figure 5D-E**), and the inhibitory SREBP1 nucleus transportation (**Figure 5F**) effects of leflunomide and teriflunomide were significantly lessened when DHODH was overexpressed in FFA-stimulated AML12 cells. These results indicate that DHODH overexpression significantly diminishes the activating AMPK signaling pathway effect of leflunomide and teriflunomide, and leflunomide and teriflunomide improve lipid metabolic disorders through regulating DHODH/AMPK signaling pathway.

### Leflunomide and teriflunomide alleviated endothelial dysfunction via regulating DHODH/AMPK signaling pathway in HUVECs

Endothelial dysfunction contributes to atherosclerosis, and disorders of lipid metabolism are important factors in endothelial cell injury. There is evidence that activation of the AMPK signaling pathway has a protective effect on vascular endothelial cells via regulating endothelial nitric oxide synthase (eNOS) 36, which promotes NO production in vascular endothelium and blood vessels thus regulating the normal function of the endothelial barrier 37-39. Since leflunomide significantly decreased plasma lipid and liver lipid accumulation in WD-fed *ApoE^-/-^* mice and FFA-stimulated AML12 cells (**Figure 1-5**), we thus investigated whether leflunomide and teriflunomide ameliorates endothelial dysfunction and protects vascular function *in vitro* and *in vivo*.

**Figure 6:**
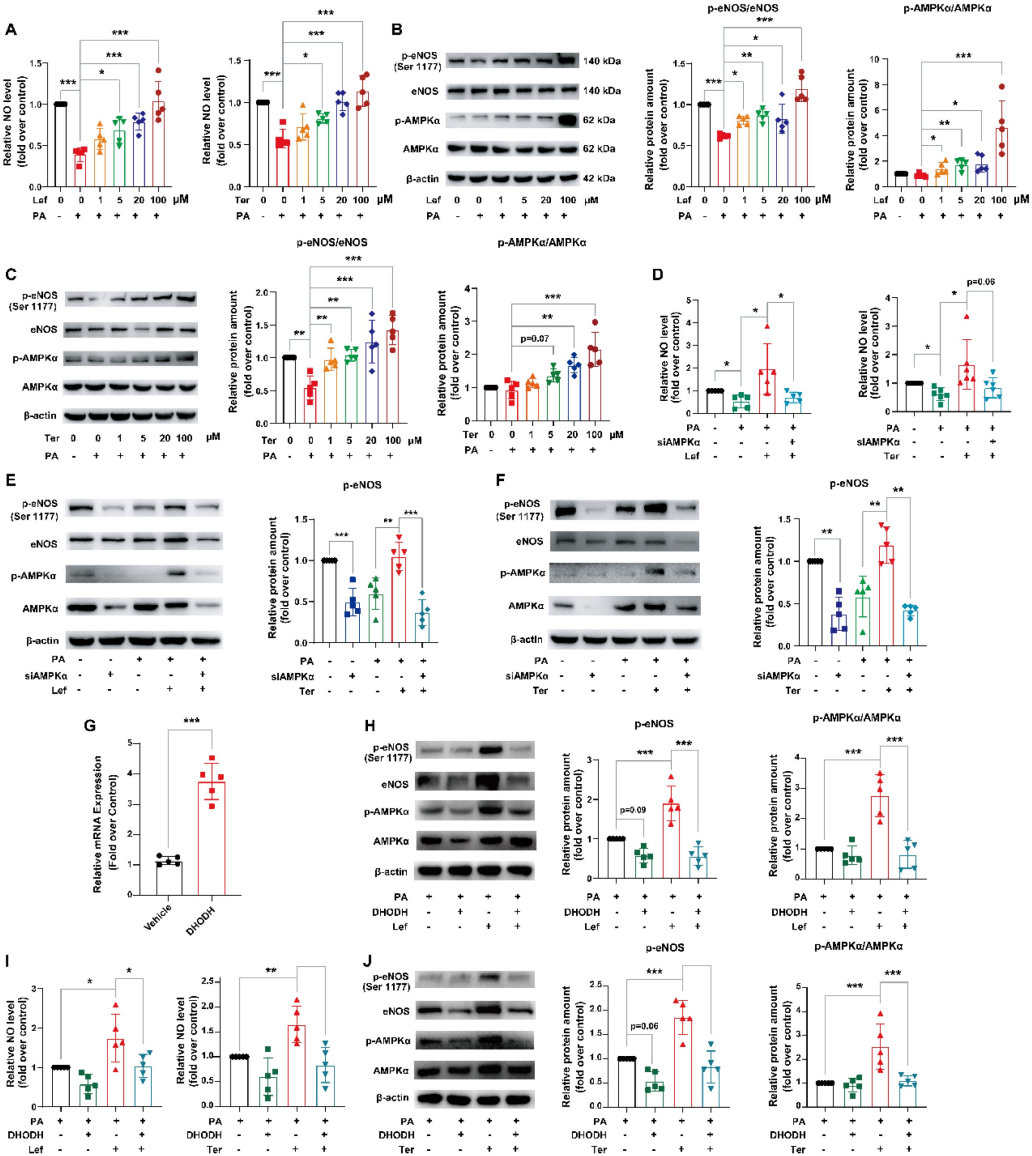
** Leflunomide and teriflunomide alleviate PA-induced endothelial dysfunction via the DHODH/AMPK/eNOS pathway. (A-C)** HUVECs with PA (500 μM) and leflunomide (0, 1, 5, 20, 100 μM) or teriflunomide (0, 1, 5, 20, 100 μM) for 18 h. **(A)** The NO production in the HUVECs with PA (500 μM) and leflunomide or teriflunomide (0, 1, 5, 20, 100 μM) for 18 h. n = 5. **(B-C)** The representative immunoblots of p-eNOS, eNOS, p-AMPKα, and AMPKα were shown, and the ratio of p-eNOS/eNOS and p-AMPKα/AMPKα were analyzed. n = 5. **(D-F)** HUVCEs were first transfected with si-control or si-AMPKα for 24 h and then were treated with PA (500 μM) and leflunomide (100 μM) or teriflunomide (100 μM) simultaneously for 18 h. **(D)** The NO production level was determined. n = 5.** (E-F)** The protein expression levels of p-eNOS, eNOS, p-AMPKα, and AMPKα were detected and the relative eNOS protein expression was quantified. n = 5. **(G)** The HUVECs were transfected with empty vector pCDNA3.1 or plasmid pCDNA3.1-DHODH, and then the mRNA levels of DHODH in HUVECs were detected using qRT-PCR. **(H-J)** HUVECs were first transfected with empty vector pCDNA3.1 or plasmid pCDNA3.1-DHODH, and then they were treated with PA (500 μM) and leflunomide (100 μM) or teriflunomide (100 μM) for 18 h. **(I)** The NO production in the cells was detected. **(H-J)** The protein levels of p-eNOS, eNOS, p-AMPKα, and AMPKα were detected and the relative expression of p-eNOS and the ratio of p-AMPKα/AMPKα were analyzed. n = 5. One-way ANOVA analysis was performed: *P < 0.05, ** P < 0.01, *** P < 0.001.

HUVECs were treated with PA to establish an endothelial dysfunction model and incubated with leflunomide or teriflunomide simultaneously. Our results showed that PA significantly decreased the NO production in HUVECs, while leflunomide and teriflunomide dose-dependently increased the NO level in PA-stimulated HUVECs (**Figure 6A**). Furthermore, leflunomide (**Figure 6B**) and teriflunomide (**Figure 6C**) increased p-eNOS and the ratios of p-AMPKα/AMPKα protein expression levels in a dose-dependent manner, indicating that they activated the AMPK signaling pathway in PA-induced HUVECs. Leflunomide also significantly increased mRNA levels of *Enos* in mouse aortic endothelial cells (**Supplementary Figure 7**). However, the increased NO production (**Figure 6D**), the protein level of the protective factor p-eNOS and p-AMPK (**Figure 6E-F**) caused by leflunomide and teriflunomide in PA-stimulated HUVECs was significantly attenuated when AMPKα was knocked down by si-AMPKα compared to that in HUVECs with si-control. Furthermore, the activation of the AMPK signaling pathway, the expression of their downstream target protein eNOS (**Figure 6H-J**) and the increased NO content (**Figure 6I**) induced by leflunomide and teriflunomide were significantly reversed when overexpression of DHODH using Flag-hDHODH plasmid in HUVECs (**Figure 6G-I**). These data suggest that leflunomide and teriflunomide alleviate endothelial dysfunction via activating AMPK and inhibiting the DHODH signaling pathway in HUVECs.

In addition, leflunomide and teriflunomide significantly reduced the protein expression of key adhesion molecules vascular cell adhesion molecule 1 (VACM1) and intercellular adhesion molecule 1 (ICAM1) in cells in a dose-dependent manner in TNFα-induced HUVECs (**Supplementary Figure 8A-D**). Furthermore, to further systematically investigate the protective effects of leflunomide on vascular function in WD-fed *ApoE^-/-^* mice, we performed RNA-Seq on the aorta of *ApoE^-/-^* mice. According to the RNA-Seq results from the aortas (**Supplementary Figure 8E-G**), leflunomide can maintain vascular homeostasis through mechanisms such as inhibition of inflammation pathways (such as chemokine signaling pathway, leukocyte transendothelial migration, cell adhesion molecules, cytokine-cytokine receptor interaction, NF-kappa B signaling pathway) and immunoregulation process (such as B cell receptor signaling pathway, T cell receptor signaling pathway, Th1 and Th2 cell differentiation and Th17 cell differentiation). All of this evidence suggests that leflunomide is effective in suppressing endothelial cell inflammatory responses and protecting endothelial cell and vascular function.

Taken together, these data indicate that leflunomide and teriflunomide attenuate endothelial dysfunction and exert a vascular protective effect via regulating the DHODH/AMPK/eNOS pathway.

## Discussion

ASCVD is the major cause of mortality and morbidity in RA patients. Leflunomide is a disease-modifying anti-rheumatic drug (DMARD) that is widely used in the treatment of RA. Therefore, it is clinically important to understand the effect of leflunomide on atherosclerosis and the underlying mechanism. In this study, we demonstrated that leflunomide treatment effectively decreased atherosclerotic plaque area both in the *en-face* aortas and aortic sinus and had a strong anti-atherosclerosis effect in WD-fed* ApoE^-/-^* mice through regulating lipid metabolism and vascular function. The mechanism studies showed that leflunomide and its active metabolite teriflunomide decreased lipid accumulation and improved endothelial dysfunction via the DHODH/AMPK signaling pathway.

Atherosclerosis is a complex disease process, and it is related to metabolic disorders of lipid and glucose 40, hypercholesterolemia 41, inflammation, insulin resistance 42, 43, endothelial dysfunction, fatty liver 44, and so on. The liver plays a vital role in controlling the circulating lipid and glucose levels, and the development of type 2 diabetes and atherosclerosis due to dysregulated hepatic fat content, insulin sensitivity, and glucose production 44. As the central mediator of energy metabolism, AMPK is vital to controlling liver lipid synthesis and fatty acid oxidation 44. Our results indicate that leflunomide activated the AMPK signaling pathway, which reduced lipid accumulation both *in vitro* and *in vivo* via increasing ACC phosphorylation and inhibiting nuclear translocation of SREBP1. Inhibiting lipid accumulation and lowering blood lipid levels are important and effective strategies for the treatment and prevention of atherosclerotic plaque formation.

Therefore, the major reason why leflunomide attenuates atherosclerosis is through activation of AMPK mediated improving lipid metabolism. In addition, the disorder of glucose metabolism is an important independent risk factor for atherosclerotic plaque development, AMPK signaling pathway plays an important role in glucose metabolism 45. Through the OGTT and ITT experiments, we observed that leflunomide could effectively improve glucose metabolism disorders in model mice, and this also exerted a positive protective effect on atherosclerosis. AMPK activation in endothelial cells led to eNOS activation and increased NO production, relieving endothelial cell dysfunction to prevent disease progression 46, 47. Furthermore, this effect was observed in PA-treated HUVECs after treatment with leflunomide or teriflunomide. Based on the significant improvements in glycolipid metabolism and endothelial dysfunction after leflunomide treatment in *ApoE^-/-^* mice, it is reasonable to believe that these effects may underlie the cardiovascular protective effects of leflunomide. Therefore, the major reason why leflunomide attenuates atherosclerosis is through activation of AMPK mediated improving liver lipid and glucose metabolism and endothelial dysfunction.

A recent study has revealed that AMPK has now been shown as a regulator in a multi-enzyme complex, named pyrimidinosome, and promotes DHODH-mediated ferroptosis defense 48. However, the mechanism and role of DHODH in the activation of AMPK and its role in the regulation of energy metabolism is not yet known. In this study, we interestingly found that overexpression of DHODH significantly reversed the effects of leflunomide and teriflunomide on regulating lipid metabolism, endothelial protection, and AMPK activation, suggesting that the activation of AMPK by leflunomide and teriflunomide was achieved through the inhibition of DHODH. Therefore, we hypothesize that DHODH may play an important role in the regulation of the AMPK signaling pathway, but more evidence is needed to unravel it. In addition, mitochondria are the primary energy source for cellular functions, and lipids and glucose can be utilized through the process of oxidative phosphorylation. The AMPK pathway regulates the various functions of mitochondria, which is the “powerhouse” of cells^44^, ^49^-^51^. DHODH, anchored in the inner mitochondrial membrane, regulates de novo pyrimidine biosynthesis and is the target of the immunosuppressive effect of leflunomide. It is speculated that leflunomide might attenuate mitochondrial dysfunction and thus improve lipid and glucose metabolism and exert anti-atherosclerotic effects via activating the AMPK signaling pathway.

Some studies have reported that leflunomide has a wide range of biological activities, including immunosuppressive, anti-inflammatory, anti-tumor, and even anti-neocoronavirus 52-54. The occurrence and development of atherosclerosis is a complex pathological process involving disorders of glucose and lipid metabolism and abnormal behavior of multiple immune cells. RNA-seq results from both liver and aorta showed that leflunomide treatment also regulates inflammation- and immune related pathways, which might be beneficial for its anti-atherosclerotic effect (**Figure 2H and Supplemental Figure 8D**). The eventual anti-atherosclerotic activities of leflunomide may be the effect of the superimposed effects of its multiple activities. Inflammation is involved in the occurrence and development of atherosclerotic plaques 28, 55, 56. The contribution of immune cells, including T cells, B cells, and mast cells, is involved in the formation of atherosclerotic plaque 57. Though leflunomide treatment had no obvious effects on the indicators (IL-1*β*, TNF*α*, and IL-6) of systemic inflammation in the plasma of the *ApoE^-/-^* mice compared to that of the WD mice (**Figure 1F**), it decreased the mRNA expression levels of *Il-1β* and *Tnfα* in peritoneal macrophages (**Supplementary Figure 1A-B**). The RNA-Seq data provided evidence (such as inhibition of the MAPK and NF-κB pathways) to support the local anti-inflammatory effect of leflunomide in the aortas and liver tissues of WD-fed *ApoE^-/-^* mice. Notably, according to the aorta RNA-Seq data, leflunomide treatment significantly affected the activity of some phenotypes of T cells compared to that in the WD group (**Supplementary Figure 8G**). In addition, many studies showed that AMPK has anti-inflammatory effects through multiple mechanisms, such as regulating macrophage-related inflammation and insulin sensitivity, under conditions of metabolic stress 44. Therefore, further studies are needed to confirm the anti-inflammatory and immune regulatory effects of immunosuppressant leflunomide in atherosclerogenesis and determine whether this effect is related to AMPK activation.

To sum up, leflunomide can improve lipid and glucose metabolism and exert vascular protection and anti-atherosclerotic effects in WD-fed *ApoE^-/-^* mice via activating AMPK and inhibiting the DHODH signaling pathway. In addition, teriflunomide, the active metabolite of leflunomide, had the same effects and mechanism on regulating lipid metabolism and endothelial cell dysfunction. Nevertheless, more trials are needed to monitor the clinical effects. Considering the cardiovascular burden in patients with immunopathology such as RA 58, 59, and the potential cardiovascular protective effect of leflunomide, it would be appropriate to use leflunomide and teriflunomide for patients with both RA and metabolic syndrome. Our study suggests a promising use of antirheumatic small-molecule drugs leflunomide and teriflunomide for the treatment of atherosclerosis and related CVDs.

## Supplementary Material

Supplementary methods, figures and table.
