# Supplementary material for: Antirheumatic drug leflunomide attenuates atherosclerosis by regulating lipid metabolism and endothelial dysfunction via DHODH/AMPK signaling pathway
Source: Int J Biol Sci. 2024 Jul 2;20(10):3725–41. doi: 10.7150/ijbs.93465 (PMC11302888; doi:10.7150/ijbs.93465)
Supplement: Supplementary file 1 — Supplementary methods, figures and table. [file ijbsv20p3725s1.pdf]

Antirheumatic small-molecule drug leflunomide attenuates atherosclerosis by regulating lipid metabolism and endothelial dysfunction via DHODH/AMPK signaling pathway

Xinhai Jiang[1, §], Weizhi Wang[1, §], Lijuan Lei[1, §], Tingting Feng[2, §], Yang Hu[3], Peng Liu[1, 4], Yining Li[1], Ren Sheng[1], Yuyan Zhang[1], Shunwang Li[1], Jing Zhang[1], Yuhao Zhang[1], Zheng-gen Jin[4], Zhuang Tian[5, *], Jiandong Jiang[1, *], Yanni Xu[1, *], Shuyi Si[1, *]

[1]State Key Laboratory of Bioactive Substances and Functions of Natural Medicines, NHC Key Laboratory of Biotechnology for Antibiotics, National Center for New Microbial Drug Screening, Institute of Medicinal Biotechnology, Chinese Academy of Medical Sciences & Peking Union Medical College (CAMS&PUMC), No.1 Tiantan Xili, Beijing 100050, China.

[2]Department of Clinical Pharmacy, Shanghai General Hospital, Shanghai Jiao Tong University School of Medicine, Shanghai, 200000, China.

[3]Pharmacy Department, Peking Union Medical College Hospital, PUMC & CAMS, Beijing, 100730, China.

[4]Department of Medicine, Aab Cardiovascular Research Institute, University of Rochester School of Medicine and Dentistry, 601 Elmwood Ave, Box CVRI, Rochester, NY 14642.

[5]Cardiology Department, Peking Union Medical College Hospital, PUMC & CAMS, No1 Shuaifuyuan, Beijing, 100730, China.

[§]These authors contributed equally to this work.

*Correspondence:

*Zhuang Tian, Email: pumchxinzang@163.com.

*Jiandong Jiang, Email: jiang.jdong@163.com.

*Yanni Xu, E-mail: xuyanniwendeng@hotmail.com.

*Shuyi Si, Tel: +86 10 63180604; Fax: +86 10 63180604; E-mail: sisyimb@hotmail.com.

Supplementary Methods

Isolation of peritoneal macrophages

ApoE$^{-/-}$ mice were treated as described in the manuscript. After euthanasia of mice, the abdomen of mice was disinfected with 75% alcohol. Then, 5 ml of PBS was injected into the mice abdominal cavity and recycled carefully. The peritoneal macrophages were collected by centrifuging at a speed of 1000 rpm/min to determine the gene expression by qRT-PCR methods.

Anti-inflammatory assay on HUVECs

HUVECs were plated in 6-well plates and treated with DMSO or different concentrations of leflunomide and teriflunomide for 18 h. Then TNFα (at a final concentration of 10 ng/mL, R&D Systems, Minneapolis, USA, Cat No. 210-TA) was added and incubated for another 6 h. The relevant protein levels of ICAM1 (Cell Signaling Technology, Cat No. 4915) and VCAM1 (Cell Signaling Technology, Cat No. 13622) were detected by Western blot.

Effect of leflunomide and teriflunomide on lipid accumulation in L02 cells

L02 cells were treated with free fatty acid (FFA, 500 μM totally; PA: OA = 1:2) and different concentrations of leflunomide and teriflunomide for 18 h simultaneously in 96-well or 6-well plates. Then the cells were stained with nile red. The relevant proteins were detected by Western blot assay.

Extraction of total RNA from mouse aortic endothelial cells

The aorta of the mouse was carefully stripped down and the adventitial fat was removed. Use the trizol solution (Thermo Fisher Scientific, Cat No.15596026CN) to repeatedly and carefully flush the lumen of the blood vessel. Then the total RNA was extracted.

Antibodies

Antibodies for Phospho-AMPKα (Thr172) (Cat No. 50081), AMPKα (Cat No. 5832), Phospho-Acetyl-CoA Carboxylase (Ser79) (Cat No. 11818), Acetyl-CoA Carboxylase (Cat No. 3676), eNOS (Cat No. 32027), Phospho-eNOS (Ser1177) (Cat No. 9570), PGC1α (Cat No. 2178), ICAM-1 (Cat No. 4915), and VCAM-1 (Cat No. 13662), were purchased from Cell Signaling Technology (Danvers, MA, USA). Antibodies for SREBP1 (Cat No. sc-365513, for immunofluorescence analysis) and HMGCR (Cat No. sc-271595) were from Santa Cruz biotechnology (Dallas, TX, USA). The antibodies for *β*-actin (Cat No. ab8226), SREBP2 (Cat No. ab30682) and FAS (Cat No.

ab82419) were from Abcam (Cambridge, United Kingdom). Antibodies for DHODH (Cat No. NBP1-86097) and SREBP1 (Cat No. NB600-582, for western blot analysis), were from Novus (Colorado, USA). Antibody for LXRα (Cat No. YT5143) was purchased from ImmunoWay Biotechnology Company (Plano, TX, USA).

Supplemental Tables and Figures

Supplemental Table 1. The qRT-PCR **primers were used in this study.**

Abca1	forward	5′-GGGTGGTGTTCTTCCTCATTAC-3′
	reverse	5′-CACATCCTCATCCTCGTCATTC-3′
Abcg1	forward	5′-TGAGCTCTTTGACCAGCTTTAT-3
	reverse	5′-AGACCCAGATCCCTCAGATAC-3′
Sr-b1	forward	5′-GGAGCATTCCTTGTTCCTAGAC-3′
	reverse	5′-CCGATGCCCTTGACAGATTT-3′
Cd-36	forward	5′-CTGGGACCATTGGTGATGAAA-3′
	reverse	5′-CACCACTCCAATCCCAAGTAAG-3
Sr-a	forward	5′-CCCTTCCTCACAGCACTAAA-3′
	reverse	5′-GCAAACACAAGGAGGTAGAGA-3′
Il-1β	forward	5′-CCACCTCAATGGACAGAATATCA-3′
	reverse	5′-CCCAAGGCCACAGGTATTT-3
Il-6	forward	5′-CTTCCATCCAGTTGCCTTCT-3′
	reverse	5′-CTCCGACTTGTGAAGTGGTATAG-3′
Tnfα	forward	5′-TTGTCTACTCCCAGGTTCTCT-3′
	reverse	5′-GAGGTTGACTTTCTCCTGGTATG-3′
Srebp2	forward	5′-TGGATGACGCAAAGGTCAA-3′
	reverse	5′-CAGGAAGGTGAGGACACATAAG-3′
Hmgcr	forward	5′-CTCATGAACGTGGTGTGTCTAT-3′
	reverse	5′-GCTCCCATCACCAAGGAATAA-3′
Enos	forward	5′-TGTGGGAGAAGATGCCAAAG-3′
	reverse	5′-GAGTCAGCCCTGGTAGTAATTG-3′

Supplemental Figure 1

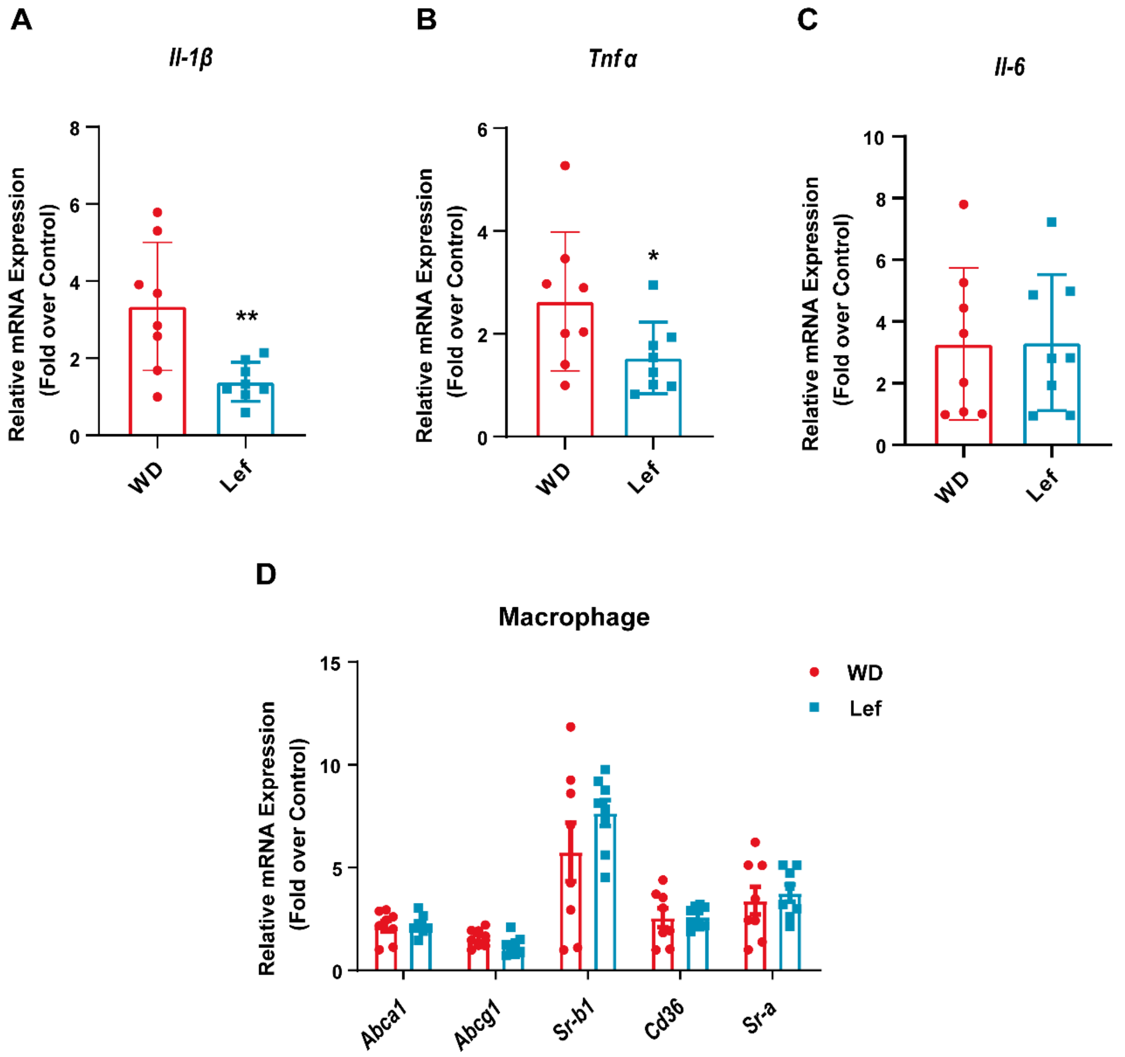

Leflunomide ameliorated inflammation in peritoneal macrophage of western diet (WD) fed *ApoE$^{-/-}$* mice, but did not affect the reverse cholesterol transport (RCT) processes. The mRNA level of **(A)** *IL-1β*, **(B)** *TNFα*, and **(C)** *IL-6* in the mice peritoneal macrophage of WD and leflunomide treated group. n = 8 per group. **(D)** The mRNA mRNA levels of *Abca1, Abcg1, Sr-b1, Cd36*, and *Sr-a* were detected with qRT-PCR. n = 8 per group. Student's *t* test analysis was performed: **P* < 0.05, ***P* < 0.01 vs WD group.

Supplemental Figure 2

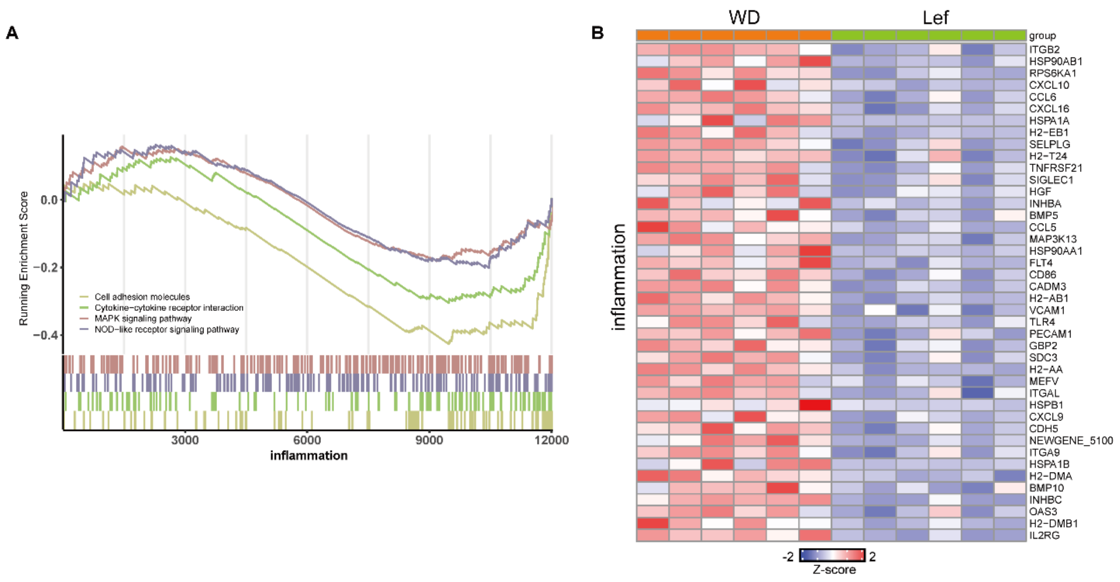

Leflunomide ameliorated liver inflammation in WD-fed *ApoE$^{-/-}$* mice based on the RNA-seq data. (A) GSEA enrichment results show the pathways related to inflammation in the RNA-Seq dataset of mice liver. **(B)** Heat map showing the representative gene expression profiles related to inflammation. *P* < 0.05. n = 6 per group.

Supplemental Figure 3

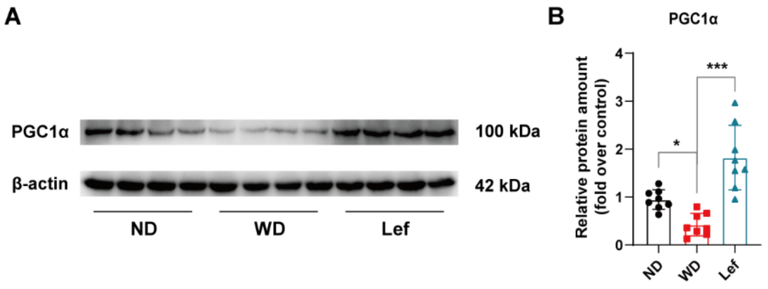

Leflunomide increased PGC1α protein levels in the liver of fed *ApoE$^{-/-}$* mice. Western blot analysis of the PGC1α in livers of *ApoE$^{-/-}$* mice in ND, WD, and leflunomide treatment group. **(A)** Representative immunoblots are shown and **(B)** the images were quantitatively analyzed with Image J software. One-way ANOVA analysis was performed: **P* < 0.05, *** *P* < 0.001. n = 4 per group.

Supplemental Figure 4

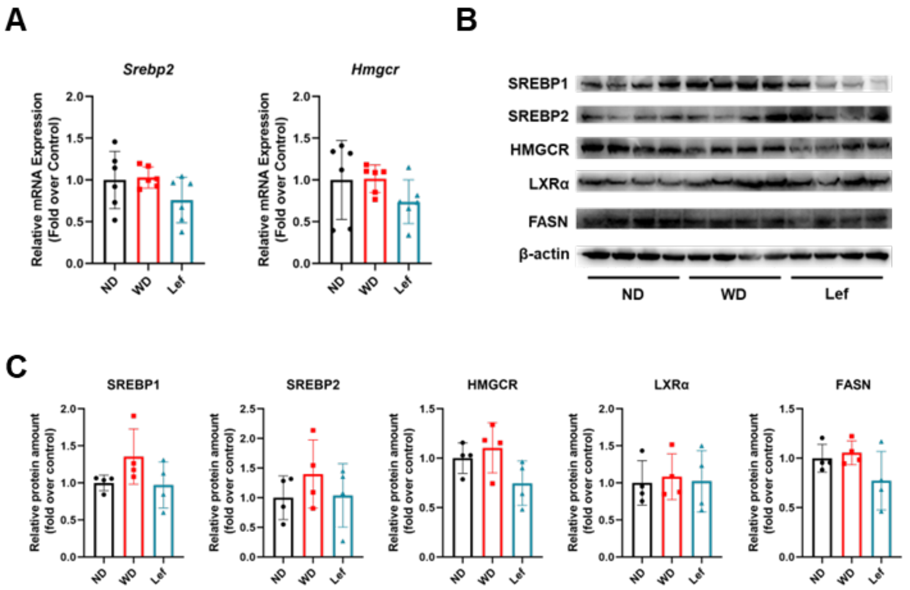

The expression levels of SREBP1, SREBP2, HMGCR LXRα and FASN in livers of *ApoE*$^{-/-}$ mice. (A) The mRNA level of *Srebp2* and *Hmgcr* in livers of *ApoE*$^{-/-}$ mice in ND, WD, and leflunomide treatment (named Lef) group, n = 6 per group. (B) Western blot analysis of the SREBP1, SREBP2, HMGCR, LXRα, and FASN in livers of *ApoE*$^{-/-}$ mice. Representative immunoblots are shown and the (C) images were quantitatively analyzed with Image J software, n = 4 per group. One-way ANOVA analysis was performed.

Supplemental Figure 5

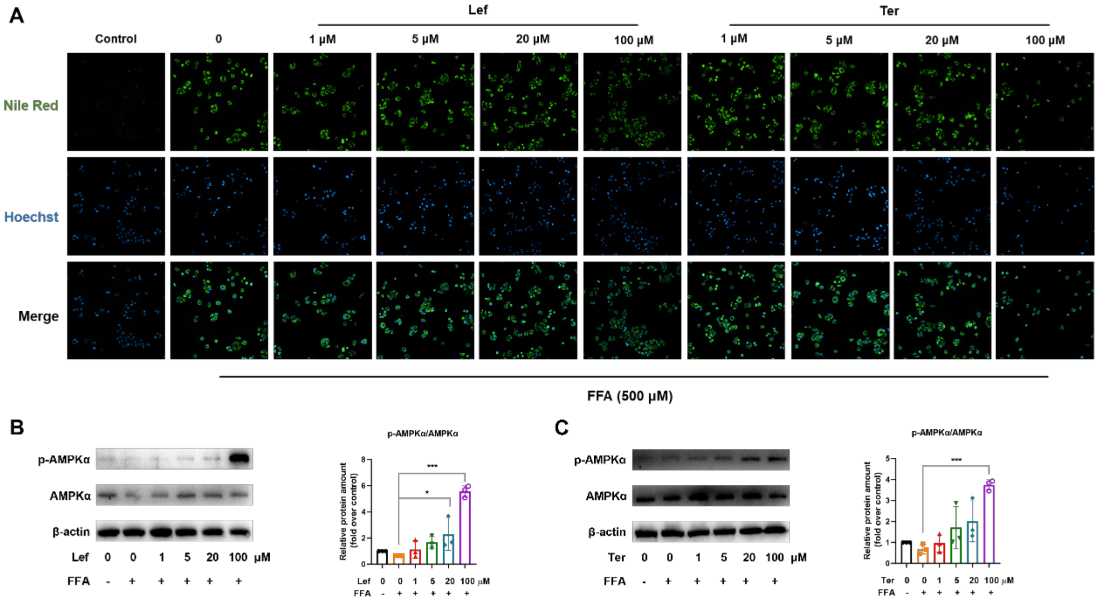

Effect of leflunomide and teriflunomide on FFA-treated L02 cells. L02 cells were treated with or without FFA (500 μM, PA: OA = 1:2) and leflunomide (0, 1, 5, 20, 100 μM) or teriflunomide (0,

1, 5, 20, 100 μM) simultaneously for 18 h. **(A)** The respective images of nile red staining. Scale bar = 20 μm. n = 3. **(B-C)** The protein level of p-AMPKα and AMPKα were detected and the representative immunoblots were shown, the ratio of p-AMPKα/AMPKα quantitatively analyzed, n = 3. One-way ANOVA analysis was performed. *$P < 0.05$, ***$P < 0.001$.

Supplemental Figure 6

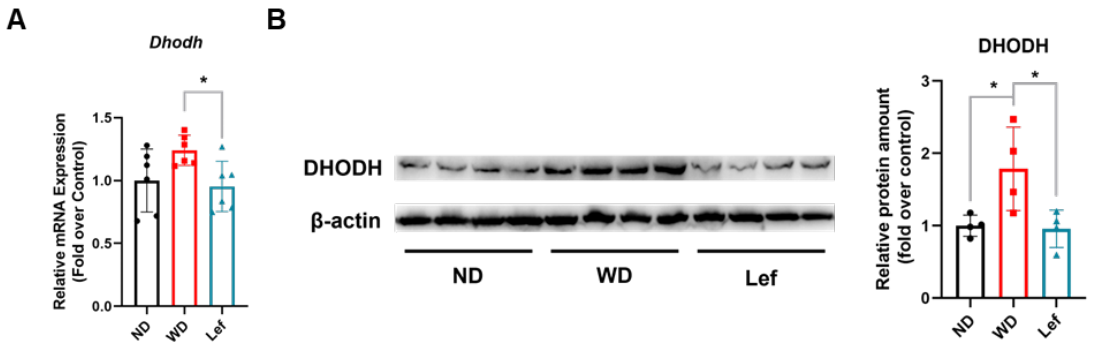

The expression levels of DHODH in *ApoE*$^{-/-}$ mice liver. (A) The mRNA level of DHODH in the liver of *ApoE*$^{-/-}$ mice based on the RNA-seq analysis. n = 6 per group. **(B)** Western blot analysis of the DHODH in livers of *ApoE*$^{-/-}$ mice. Representative immunoblots were shown and the images were quantitatively analyzed with Image J software. n = 4 per group. One-way ANOVA analysis was performed, *$P < 0.05$.

Supplemental Figure 7

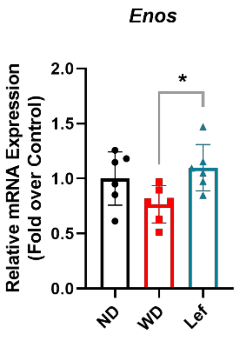

The mRNA level of *Enos* in aortic endothelial cells of *ApoE*$^{-/-}$ mice. The mRNA level of *Enos* in aortic endothelial cells of *ApoE*$^{-/-}$ mice with qRT-PCR One-way ANOVA analysis was performed, *$P < 0.05$, n = 6 per group.

Supplemental Figure 8

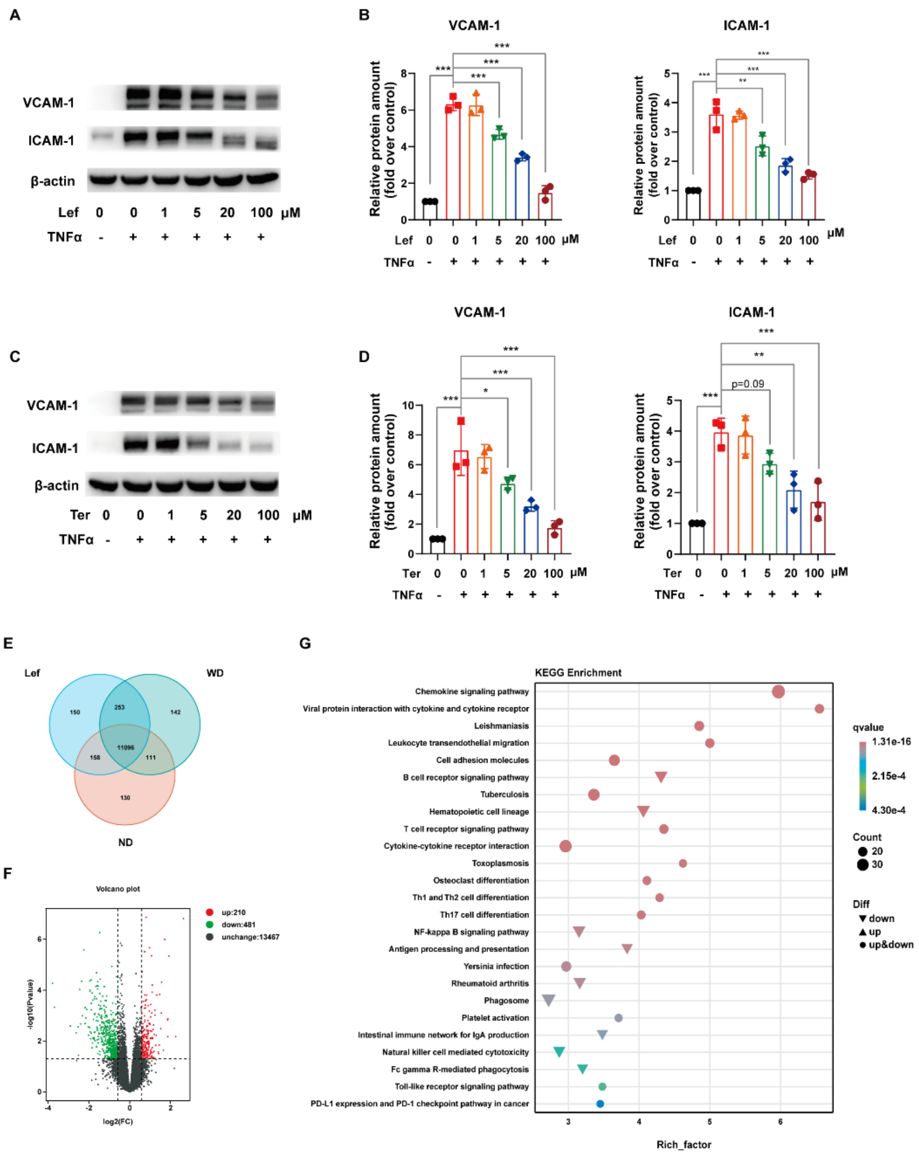

Leflunomide improves vascular function homeostasis in WD-fed *ApoE*$^{-/-}$ mice. (A-D) HUVECs were pretreated with DMSO or leflunomide or teriflunomide (0, 1, 5, 20, 100 μM) for 18h, TNFα (final concentration 10 ng/ml) was then added for a further 6h. Western blots were performed to examine the protein expression levels of VCAM-1 and ICAM-1 in treated HUVECs. Image J software was used, and representative immunoblots were shown. n = 5. **(E)** Venn diagram based on RNA-seq results from the aorta of ND, WD and leflunomide treatment groups *ApoE*$^{-/-}$ mice. n = 6 per group. **(F)** Volcano map of differentially expressed genes (DGEs) (fold change ≥ 1.5 and *P* value < 0.01, WD vs Lef) in RNA-seq results from the aorta of ND, WD and leflunomide treatment groups *ApoE*$^{-/-}$ mice. n = 6 per group. **(G)** KEGG enrichment analysis DGEs between WD and

leflunomide treated group mice aorta, $P < 0.05$, n = 6 per group. One-way ANOVA analysis was performed: *$P < 0.05$, ** $P < 0.01$, *** $P < 0.001$.
